# Deep Learning in Glaucoma Detection and Progression Prediction: A Systematic Review and Meta-Analysis

**DOI:** 10.3390/biomedicines13020420

**Published:** 2025-02-10

**Authors:** Xiao Chun Ling, Henry Shen-Lih Chen, Po-Han Yeh, Yu-Chun Cheng, Chu-Yen Huang, Su-Chin Shen, Yung-Sung Lee

**Affiliations:** 1 Department of Ophthalmology, Chang Gung Memorial Hospital, Linkou, Taoyuan 333, Taiwan; lingxiaochun1991@gmail.com (X.C.L.);; 2 Graduate Institute of Clinical Medical Sciences, Chang Gung University, Taoyuan 333, Taiwan; 3 College of Medicine, Chang Gung University, Taoyuan 333, Taiwan; 4 Department of Ophthalmology, New Taipei Municipal Tucheng Hospital, New Taipei 236, Taiwan

**Keywords:** glaucoma, deep learning, artificial intelligence, fundus photography, optical coherence tomography, diagnosis, progression

## Abstract

**Purpose:** To evaluate the performance of deep learning (DL) in diagnosing glaucoma and predicting its progression using fundus photography and retinal optical coherence tomography (OCT) images. **Materials and Methods:** Relevant studies published up to 30 October 2024 were retrieved from PubMed, Medline, EMBASE, Cochrane Library, Web of Science, and ClinicalKey. A bivariate random-effects model was employed to calculate pooled sensitivity, specificity, positive and negative likelihood ratios, and area under the receiver operating characteristic curve (AUROC). **Results:** A total of 48 studies were included in the meta-analysis. DL algorithms demonstrated high diagnostic performance in glaucoma detection using fundus photography and OCT images. For fundus photography, the pooled sensitivity and specificity were 0.92 (95% CI: 0.89–0.94) and 0.93 (95% CI: 0.90–0.95), respectively, with an AUROC of 0.90 (95% CI: 0.88–0.92). For the OCT imaging, the pooled sensitivity and specificity were 0.90 (95% CI: 0.84–0.94) and 0.87 (95% CI: 0.81–0.91), respectively, with an AUROC of 0.86 (95% CI: 0.83–0.90). In predicting glaucoma progression, DL models generally showed less robust performance, with pooled sensitivities and specificities ranging lower than in diagnostic tasks. Internal validation datasets showed higher accuracy than external validation datasets. **Conclusions:** DL algorithms achieve excellent performance in diagnosing glaucoma using fundus photography and OCT imaging. To enhance the prediction of glaucoma progression, future DL models should integrate multimodal data, including functional assessments, such as visual field measurements, and undergo extensive validation in real-world clinical settings.

## 1. Introduction

Glaucoma leads to irreversible vision impairment across the globe, with projections indicating its impact on over 111 million individuals by 2040 [1]. Due to the increasing disease burden of glaucoma worldwide, the accurate detection and diagnosis of glaucoma are essential. The diagnosis of glaucoma involves multiple diagnostic modalities, with the intraocular pressure (IOP) measurement being an important risk factor, alongside assessments such as optic disc evaluation, optical coherence tomography (OCT), and Humphrey’s visual field analysis [2]. In particular, cases with normal tension glaucoma require a comprehensive evaluation of optic nerve structure and visual field function, as IOP may fall within the normal range [3]. Therefore, widespread glaucoma evaluation by certified clinicians is resource-consuming and skill-dependent and, sometimes, inconsistent [4,5].

Deep learning (DL) is a subset of artificial intelligence (AI) that involves training neural networks to learn patterns and make predictions based on large datasets [6]. It can learn intricate features directly from raw data, making it suitable for tasks involving large and complex datasets, such as medical image analysis [6]. DL can be utilized in glaucoma detection via analyzing fundus photographs or OCT scans [7] by recognizing subtle structural changes indicative of glaucoma, such as optic nerve head morphology (cup-to-disc ratio) or retinal nerve fiber layer thickness [8]. Additionally, with longitudinal visual field and clinical data, DL models may extract spatiotemporal features that may provide better assessments of glaucoma progression [9].

Despite promising results from various studies, the translation of AI into clinical practice remains limited [10,11]. Conducting an updated meta-analysis will help consolidate existing evidence and identify factors that influence the performance of AI. This is important now, as recent advancements in DL models have significantly improved their ability to analyze complex imaging data, offering enhanced accuracy and efficiency in glaucoma detection and progression prediction [12,13]. Additionally, the growing availability of large, annotated datasets [14,15] provides an unprecedented opportunity to train and validate these models, necessitating a consolidation of the evidence to evaluate their generalizability and clinical applicability.

Previous meta-analyses have explored the use of AI in glaucoma diagnosis [10,16,17], highlighting the generally strong performance of AI algorithms in detecting glaucoma using fundus or OCT imaging. However, these studies have primarily focused on diagnostic accuracy, with limited analysis on glaucoma progression. This gap in the literature has guided our study design, motivating us to evaluate the potential of DL algorithms to not only diagnose glaucoma but also assess its progression.

Our primary aim is to evaluate the overall recent performance of DL algorithms in diagnosing glaucoma and identifying its progression using fundus photography and OCT, as these are the most commonly available apparatuses in clinical programs worldwide. Through this evaluation, we seek to further understand the potential utility of AI in real-world glaucoma assessment and management.

## 2. Materials and Methods

We describe the protocols, strategies, and analytical methods used to identify and assess studies related to the application of DL in glaucoma detection and progression prediction here. This section outlines the systematic approach to study selection, data collection, and quality assessment, followed by the analytical procedures for synthesizing results.

### 2.1. Protocol and Registration

The study was conducted in accordance with the Preferred Reporting Items for Systematic Reviews and Meta-Analysis of Diagnostic Test Accuracy (PRISMA-DTA) extension guidelines [18]. Institutional Review Board (IRB) approval and informed consent were not required. This study protocol was registered in the International Prospective Register of Systematic Reviews (PROSPERO ID: CRD42024510390). All data were extracted from past publications, and all research adhered to the Declaration of Helsinki. These measures helped establish the transparency and reproducibility of the research process.

### 2.2. Search Strategy for Identifying Studies

Relevant studies published through 30 October 2024 regarding the utilization of DL or AI in glaucoma detection, diagnosis, or progression prediction were retrieved from PubMed, Medline, EMBASE, Cochrane Library, Web of Science (WoS), and ClinicalKey.

Search keywords comprised a combination of terms associated with glaucoma—“Glaucoma”, “Open-angle glaucoma”, and “Glaucomatous optic neuropathy”—with the following keywords associated with artificial intelligence: “Deep learning”, “Machine learning”, and “Artificial intelligence”. Terms involving outcomes were also used, such as “Glaucoma diagnosis” and “Glaucoma Progression”. No language or time restrictions were applied. The detailed search strategy is provided in the Supplemental Material.

### 2.3. Eligibility Criteria and Study Selection

The study selection, data collection, and quality assessment were carried out by the authors XCL and YSL. After removing duplicates, titles and abstracts were screened for eligibility based on the publication type and reported outcomes. The eligible studies included full-length journal articles presenting findings on the performance of DL in distinguishing glaucoma from the normal population using automated analyses of fundus or OCT images. In terms of progression predictions, the eligible studies were those that reported glaucoma progression in terms of categorical outcomes such as “no progression” or “progression/conversion”. Articles were excluded if they did not assess primary open-angle glaucoma. Case reports, guidelines, conference papers, letters, editorials, and review articles were excluded. Additionally, studies that did not provide primary outcomes related to predicting glaucoma onset, detecting glaucoma progression, or distinguishing glaucoma from other ocular diseases were omitted from consideration.

### 2.4. Data Collection

Data collection was conducted during the full-text review. The extracted variables from each study, when available, included the first author’s name, publication year, country of origin, type of images (fundus or OCT), primary DL classifier, total size of image data, data sources for training and validation, total size of testing data, ground truth information, validation type (internal or external), as well as the sensitivity, specificity, and area under the receiver operating characteristic (AUROC) curve of the most effective DL algorithm outcomes obtained from each validation or testing dataset. We thoroughly reviewed each full-text article included in the study to identify irrelevant outcomes and data insufficiency, defined as the absence of adequate information required to construct a 2 × 2 contingency table for the testing results.

### 2.5. Risk of Bias Assessment

We evaluated the quality of each incorporated study utilizing the Quality Assessment of Diagnostic Accuracy Studies 2 (QUADAS-2) tool. QUADAS-2 encompasses four key domains for assessment: patient selection, index test, reference standard, and flow and timing. The risk of bias for each domain was rated as low, high, or unclear based on the information available in each study. Studies with low risk across most domains were considered methodologically robust, while high or unclear risks indicated potential bias or gaps in reporting. Applicability concerns were similarly assessed to determine the relevance of the study populations, diagnostic tests, and reference standards to clinical practice. The findings from the QUADAS-2 assessments were visualized using traffic-light plots to summarize the proportion of studies with low, high, or unclear risk in each domain. Additionally, we assessed the general applicability of the studies to the population of interest, ensuring that the findings would be relevant to real-world clinical practice.

### 2.6. Analysis and Data Synthesis

A bivariate random-effects model was utilized to perform a meta-analysis on the diagnostic and progression prediction performance of DL in glaucoma. The bivariate method was used to model logit-transformed sensitivity and specificity together, addressing the negative correlation resulting from different threshold settings across various studies. Data from different image types (fundus and OCT) were analyzed separately. When multiple DL algorithms or feature selections were tested, the best-performing DL testing results of each dataset were chosen. Multiple datasets from a study were documented separately and were considered independent data for the meta-analysis.

We calculated the pooled sensitivity, specificity, positive and negative likelihood ratios (LRs), and AUROC, with the results presented through hierarchical summary receiver operating characteristic (sROC) curve plots. The sROC curves were generated using a bivariate random-effects model, which accounts for the correlation between sensitivity and specificity across the studies. This approach enables the estimation of a summary ROC curve that reflects the overall performance of the models while addressing the inter-study heterogeneity. We further quantified the variation of effect size due to inter-study heterogeneity with forest plots, providing visual insights into study-level variability and the pooled estimates.

To assess the risk of publication bias, Deeks’ funnel plots were used. We used Egger’s test, which applies a rank correlation to test for publication bias; thus, it is less likely to produce false positives. We employed DeLong’s test, a non-parametric statistical method, for comparing the calculated performance metrics. Given the multiple comparisons conducted across several metrics and datasets, we applied a Bonferroni correction to adjust for the increased risk of type I errors. A data synthesis and analysis were performed using RevMan, Version 5.4 (Cochrane). A 2-sided *p*-value of 0.05 indicated statistical significance.

## 3. Results

The results of this meta-analysis are structured into distinct subsections to provide clarity into the steps of the analysis, from the initial search to the final performance outcomes of the DL models.

### 3.1. Search Results

**Figure 1:**
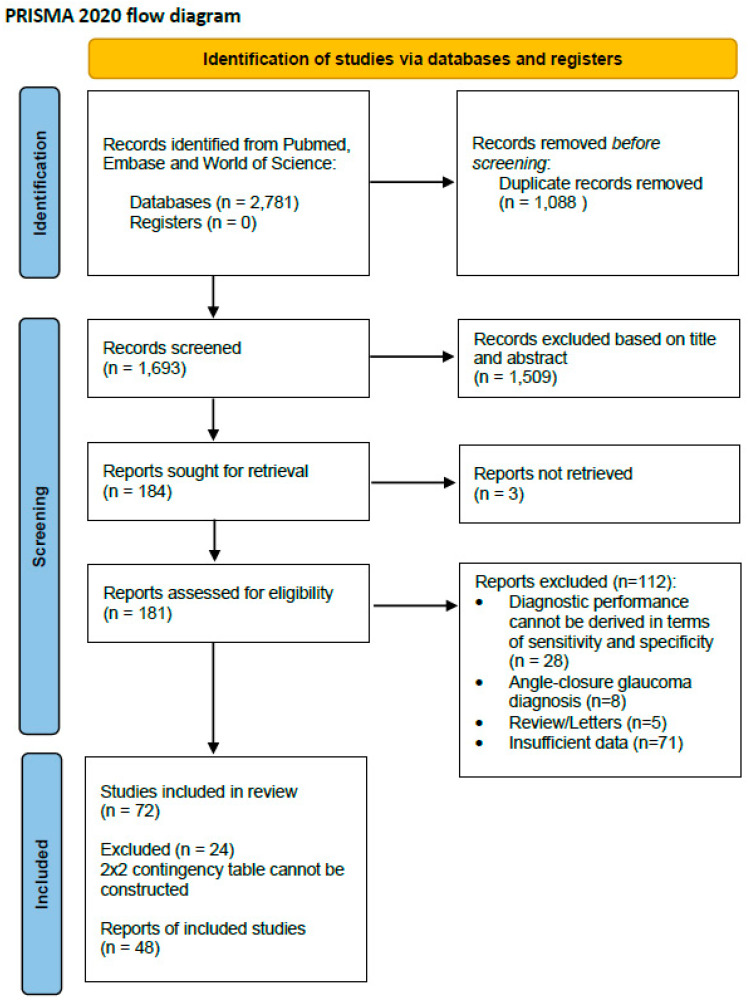
PRISMA 2020 flow diagram for systematic reviews and meta-analysis.

The literature search yielded 2781 records in total from PubMed, EMBASE, and Web of Science. After excluding 1088 duplicates, the titles and abstracts of 1693 studies were screened for eligibility. A total of 1509 records were excluded due to non-relevant titles and abstracts. Three reports could not be retrieved in full even after requesting them from the authors. Following screening, a total of 112 records were excluded, including 28 due to insufficient data to derive the sensitivity and specificity, 8 because they focused on angle-closure glaucoma, 5 because they were letters or reviews, and 71 due to inaccessible or insufficiently reported data. Finally, 72 studies were included for a systematic review, and among these, 48 were integrated for a data analysis through a meta-analysis, as a 2 × 2 contingency table could not be constructed for the other studies. The Preferred Reporting Items for Systematic Reviews and Meta-Analyses (PRISMA) flowchart of the study selection is presented in Figure 1.

### 3.2. Study Characteristics

The characteristics of all included studies are summarized in Table 1 and Table 2. A total of 55 datasets from 42 studies used fundus or OCT imaging DL algorithms to diagnose glaucoma (Table 1), while 8 datasets from 7 studies used these algorithms in glaucoma progression prediction (Table 2). Across all studies evaluating diagnostic performance, 2,229,810 fundus images and 51,386 OCT images were used for training, while fundus images from 194,259 subjects and OCT images from 17,882 subjects were used for testing. For the studies evaluating glaucoma progression, a total of 22,422 fundus and 86,123 OCT images were used for training, while fundus images from 841 subjects and 34,847 OCT images were used for testing.

Out of the 63 datasets from 48 studies included, 22 datasets (34.92%) utilized external testing to evaluate AI performance in diagnosis or progression prediction, which means that the tested datasets were different from the training datasets. The included studies employed a diverse range of DL architectures. Notably, convolutional neural networks (CNNs) were a common choice (58.33%) due to their proven efficacy in image-based diagnostics. Other ensemble methods for image analysis included modules such as XGBoost, random forest, gradient boosting, and many more [19,20,21,22,23,24,25,26,27,28,29,30,31,32,33,34,35,36,37,38,39,40,41,42,43,44,45,46,47,48,49,50,51,52,53,54,55,56,57,58,59,60,61,62,63,64,65,66].

#### 3.2.1. Risk of Bias and Applicability of Included Studies

The graphical results of the quality assessment are presented in Supplementary Figures S1 and S2. A majority of studies had a low risk of bias and concerns regarding applicability for the reference standard, according to the QUADAS-2 tool.

About 75% of the included studies demonstrated a low risk of bias in patient selection, indicating that the majority utilized appropriate inclusion and exclusion criteria to ensure representative study populations. A small proportion of the studies were classified as unclear or high risk of bias due to inadequate reporting of recruitment strategies or the use of convenience samples rather than random or consecutive sampling.

The index test domain assessed the risk of bias associated with the DL models used in the studies. Less than 25% of the studies were classified as (unclear) due to insufficient details regarding how the DL models were validated or thresholds were pre-specified. The applicability concerns were similarly low across most studies, indicating that the DL models were suitable for the intended diagnostic tasks.

The reference standard domain was evaluated to ensure that the criteria used to confirm glaucoma diagnoses or progression were reliable and unbiased. A large proportion of studies (>80%) had a low risk of bias, as they employed well-established diagnostic methods, such as clinical evaluations by glaucoma specialists or well-established imaging techniques.

The flow and timing domain was assessed for potential bias due to deviations in the timing between the index test and reference standard or issues related to participant attrition. A considerable number (about 20%) were classified as unclear due to insufficient reporting of the timing between tests or missing data.

#### 3.2.2. Publication Bias

We utilized Deeks’ funnel plot asymmetry test to evaluate publication bias and to illustrate asymmetry in the funnel plot. From the funnel plot, shown in Supplementary Figure S3, the studies in this analysis appeared to be symmetrically distributed around the regression line, indicating no apparent publication bias. Egger’s test for overall publication bias revealed *p* = 0.55 (Supplementary Figure S3). Publication bias is considered significant if *p* < 0.10. This result suggested that the included studies were not disproportionately skewed toward those reporting more favorable or positive findings.

### 3.3. Overall Performance of DL Using Fundus Photography in Glaucoma Detection

**Figure 2:**
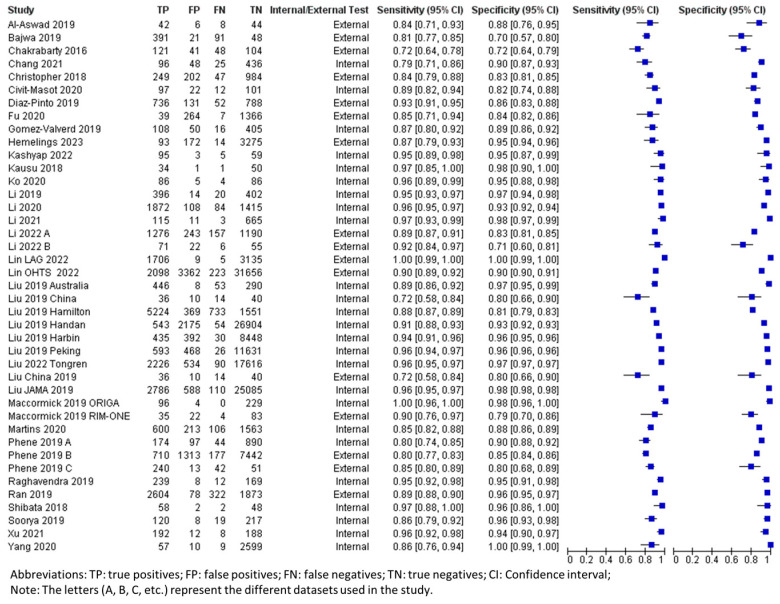
Forest plot for sensitivities and specificities of glaucoma diagnosis with DL using fundus photography [19,21,22,23,24,25,26,27,28,29,30,31,33,37,38,39,40,41,43,44,45,46,49,50,51,52,53,55,58,59].

**Figure 3:**
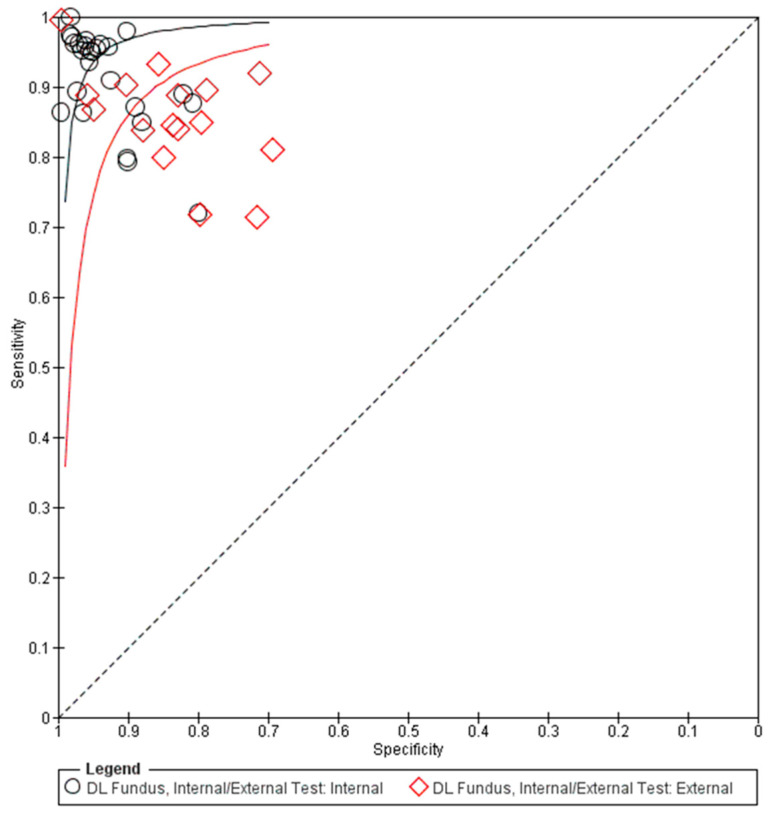
Summary receiver operating characteristic (sROC) of all included data using fundus photographs in glaucoma diagnosis. DL: deep learning.

The pooled summary of DL performance in glaucoma detection using fundus photography is shown in Table 3. Forest plots detailing the sensitivities and specificities of all datasets are shown in Figure 2. The corresponding summary receiver operating characteristic (sROC) plots for diagnostic performance are shown in Figure 3. A total of 42 datasets were plotted (data size *n* = 194,259). The sROC curves were plotted separately for internal validation (black) and external validation (red) to illustrate the distinction between internal and external validation of the DL algorithms in the diagnosis. The internal testing generally showed higher accuracy (higher sensitivity and specificity) than the external testing, which revealed higher variability among the datasets, as shown in the sROC plot (Figure 3).

By considering all data for the use of fundus photography in the DL algorithm (Table 3), the pooled mean sensitivity, specificity, positive likelihood ratio, negative likelihood ratio, and area under the receiver operating characteristic curve (AUROC) were 0.92 (95% CI 0.89–0.94), 0.93 (95% CI 0.90–0.95), 12.99 (95% CI 9.23–18.30), 0.09 (95% CI 0.07–0.12), and 0.90 (95% CI 0.88–0.92), respectively. From the internally validated datasets (n = 124,552), the pooled mean sensitivity, specificity, positive likelihood ratio, negative likelihood ratio, and AUROC were 0.93 (95% CI 0.91–0.95), 0.95 (95% CI 0.93–0.97), 18.77 (95% CI 13.09–26.91), 0.07 (95% CI 0.05–0.10), and 0.91 (95% CI 0.90–0.93), respectively. The externally validated datasets (n = 69,707) revealed a generally decreased performance as follows: the pooled mean sensitivity, specificity, positive likelihood ratio, negative likelihood ratio, and AUROC were 0.86 (95% CI 0.82–0.93), 0.88 (95% CI 0.80–0.93), 7.259 (95% CI 4.14–12.73), 0.13 (95% CI 0.08–0.22), and 0.88 (95% CI 0.86–0.91), respectively.

### 3.4. Overall Performance of DL Using OCT in Glaucoma Detection

**Figure 4:**
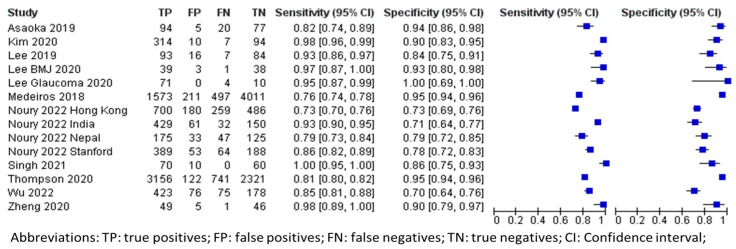
Forest plot for sensitivities and specificities of glaucoma diagnosis with DL using OCT imaging [20,32,34,35,36,47,48,54,56,57,60].

**Figure 5:**
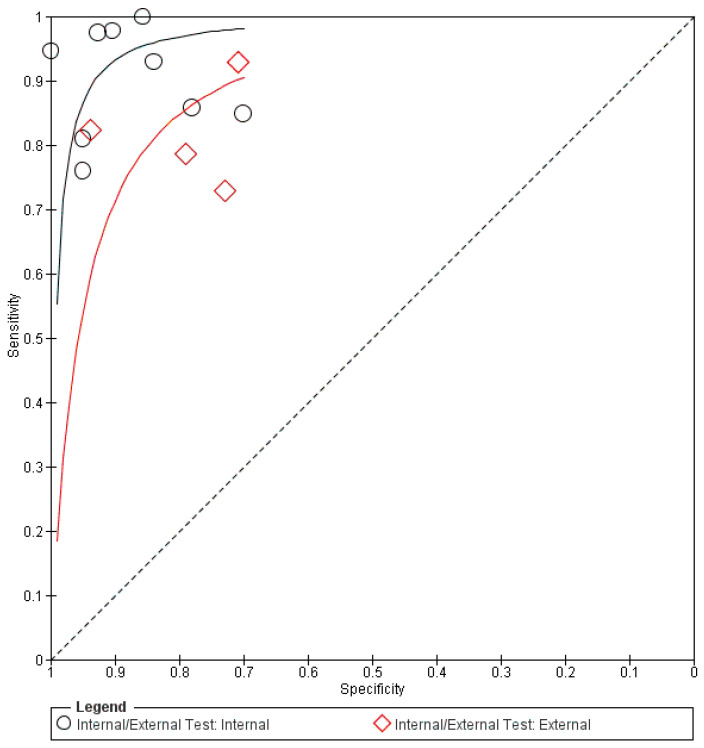
Summary receiver operating characteristic (sROC) of all included data using OCT imaging in glaucoma diagnosis. DL: deep learning.

In terms of OCT imaging, the aggregated data (n = 17,882) from all datasets are also shown in Table 3. The forest plots and sROC curves for both internal (black) and external (red) datasets are shown in Figure 4 and Figure 5, respectively. Similar to the fundus photography, the externally tested performance of the AI algorithm using OCT for glaucoma diagnosis has lower overall accuracy compared to the internal testing, as shown in Figure 5.

The pooled mean sensitivity, specificity, positive likelihood ratio, negative likelihood ratio, and AUROC for the overall OCT images in glaucoma diagnosis were 0.90 (95% CI 0.84–0.94), 0.87 (95% CI 0.81–0.91), 6.87 (95% CI 4.57–10.33), 0.11 (95% CI 0.07–0.19), and 0.86 (95% CI 0.83–0.90), respectively. The internal testing (n = 15,009) revealed much better diagnostic performance than the external testing. The pooled mean sensitivity, specificity, positive likelihood ratio, negative likelihood ratio, and AUROC for the internal testing were 0.93 (95% CI 0.85–0.96), 0.93 (95% CI 0.85–0.96), 8.46 (95% CI 5.23–13.68), 0.08 (95% CI 0.04–0.17), and 0.89 (95% CI 0.85–0.93), respectively. In contrast, the external testing (n = 2873) showed pooled mean sensitivity, specificity, positive likelihood ratio, negative likelihood ratio, and AUROC of 0.83 (95% CI 0.73–0.90), 0.80 (95% CI 0.68–0.88), 4.20 (95% CI 2.56–6.89), 0.21 (95% CI 0.13–0.34), and 0.80 (95% CI 0.75–0.86), respectively.

### 3.5. Overall Performance of DL Using Fundus Photography/OCT in Glaucoma Progression Prediction

**Figure 6:**
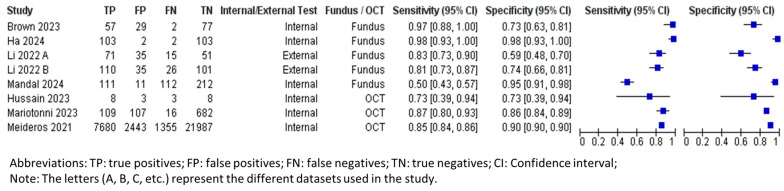
Forest plot for sensitivities and specificities of glaucoma progression predictions with DL using fundus photography/OCT imaging [37,61,62,63,64,65,66].

**Figure 7:**
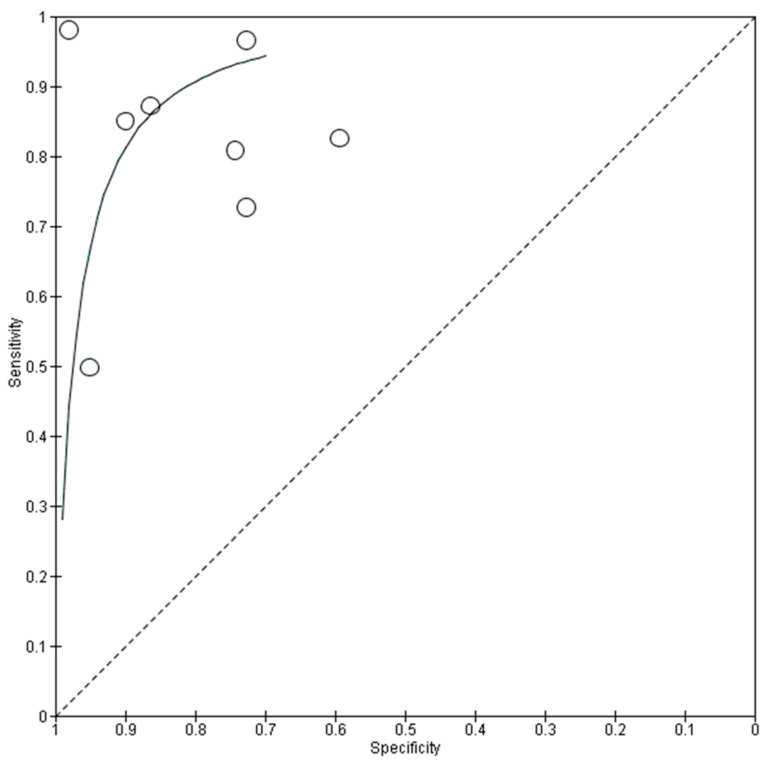
Summary receiver operating characteristic (sROC) of all included data using fundus photography/OCT imaging in progression predictions.

Table 4 summarizes the overall performance of both fundus photography and OCT in DL for glaucoma progression predictions. The number of tested eyes in each dataset was used for calculating the performance metrics, instead of data size, ensuring a more accurate representation of the longitudinal nature of the data, as each eye could contribute multiple images for testing. The forest plots and sROC curves for glaucoma progression predictions are shown in Figure 6 and Figure 7, respectively.

The pooled mean sensitivity, specificity, positive likelihood ratio, negative likelihood ratio, and AUROC for all fundus photography (tested patients, n = 841) were 0.89 (95% CI 0.78–0.95), 0.77 (95% CI 0.64–0.87), 3.88 (95% CI 2.31–6.51), 0.14 (95% CI 0.07–0.30), and 0.91 (95% CI 0.85–0.97), respectively. The internally tested datasets (tested patients, n = 375) had a pooled mean sensitivity, specificity, positive likelihood ratio, negative likelihood ratio, and AUROC of 0.95 (95% CI 0.82–0.99), 0.88 (95% CI 0.60–0.97), 7.87 (95% CI 1.97–31.48), 0.06 (95% CI 0.02–0.23), and 0.94 (95% CI 0.84–1.00), respectively. Similar to the diagnostic performance, the external testing for progression predictions (tested patients, n = 466) produced a lower performance, with pooled mean sensitivity, specificity, positive likelihood ratio, negative likelihood ratio, and AUROC at 0.81 (95% CI 0.77–0.85), 0.67 (95% CI 0.51–0.79), 2.44 (95% CI 2.18–2.73), 0.28 (95% CI 0.23–0.34), and 0.88 (95% CI 0.84–0.92), respectively.

The pooled mean sensitivity, specificity, positive likelihood ratio, negative likelihood ratio, and AUROC for OCT in predicting glaucoma progression were 0.74 (95% CI 0.51–0.89), 0.93 (95% CI 0.88–0.96), 10.09 (95% CI 7.60–13.39), 0.28 (95% CI 0.14–0.57), and 0.90 (95% CI 0.84–0.95), respectively. Currently, only the internally tested databases have been published in the literature and are included in this meta-analysis. The pooled specificity is significantly higher than the pooled sensitivity, indicating that the OCT AI algorithm is more effective at correctly identifying patients who will not experience glaucoma progression.

### 3.6. Comparative Analyses Between Performance Metrics (Sensitivity, Specificity, Positive and Negative Likelihood Ratios, and AUROC)

Each performance metric between the subgroups was statistically compared via DeLong’s test with Bonferroni correction (Supplementary Table S1). From the comparative analysis, fundus imaging DL demonstrated significantly lower sensitivity (*p* < 0.05) for progression predictions when tested on external datasets compared to its performance in glaucoma diagnosis when tested internally. This highlights the challenge of generalizing progression prediction models, particularly across diverse external databases. Fundus imaging DL also consistently achieved the highest specificity in internal databases, underscoring its robustness in accurately excluding non-glaucomatous cases.

OCT demonstrated a significantly higher positive likelihood ratio compared to fundus imaging in progression predictions. This indicates that OCT was more effective in confirming disease progression when an abnormal test result was observed. The negative likelihood ratio for fundus imaging was consistently lower in both glaucoma diagnosis and progression predictions compared to OCT.

The internally tested fundus imaging showed statistically significant superiority (*p* < 0.05) in AUROC scores for glaucoma diagnosis compared to the externally tested OCT. This highlights the strength of fundus imaging in achieving high diagnostic accuracy in controlled settings.

## 4. Discussion

This study evaluated recent performances of DL algorithms in diagnosing glaucoma and predicting glaucoma progression using fundus photography and OCT images. Overall, DL models achieved good diagnostic performance, as evidenced by high sensitivity, specificity, and AUROC. The glaucoma progression prediction test results with DL using fundus and OCT imaging were less robust, probably due to the limited availability of longitudinal data, the complexity of structural features, and challenges in DL training without functional assessment integration. Throughout all studies, the internal testing of both diagnostic and progression prediction DL performance produced better results compared to the external testing.

In comparison to previous reviews, this analysis demonstrated that DL algorithms based on fundus and OCT imaging modalities attained good accuracy in glaucoma diagnosis (AUC 96% and AUC 95%, respectively) and progression prediction (AUC 91% and AUC 90%, respectively). A recent study by Wu et al. [16] on machine learning algorithms showed good performance in glaucoma diagnosis. Although Chaurasia et al. [10] demonstrated in their meta-analysis that AI algorithms for both fundus and OCT imaging modalities showed comparable accuracy (96.2% AUC and 96.0% AUC, respectively) for glaucoma detection, another study by Murtagh et al. [67] reported a lower AUC (92.3%) on OCT images, which was similar to our study.

Sensitivity and specificity are key metrics for evaluating diagnostic algorithms. Sensitivity reflects the ability to identify true positive cases, while specificity measures the accuracy in identifying true negatives. The relative importance of these metrics depends on the clinical context. For glaucoma diagnosis, fundus imaging achieves a sensitivity of 92% and a specificity of 93%, while OCT achieves a sensitivity of 90% and a specificity of 87%. These high values highlight the robustness of the models in detecting glaucoma. However, sensitivity and specificity differ for glaucoma progression predictions. Fundus imaging shows a sensitivity of 89% but a lower specificity of 77%, whereas OCT demonstrates a sensitivity of 74% and a higher specificity of 93%. The higher specificity of OCT in glaucoma progression predictions reflects that the structural markers in OCT are well defined and quantifiable with time, thus making OCT more effective at identifying true negative cases (no progression).

There are several reasons why DL algorithms using OCT showed lower sensitivity, specificity, and overall AUC compared to fundus imaging in diagnosis. Firstly, studies included in this meta-analysis showed that OCT data had comparatively fewer training samples than fundus imaging, which may result in poorer generalization. Additionally, published studies often highlight that while OCT provides valuable depth information for certain aspects of glaucoma assessments, fundus images remain more straightforward for automated detection tasks due to their consistency, simplicity, and greater prevalence in large datasets used for model training [68]. Notably, fundus imaging is widely used in large screening programs, leading to more standardized protocols from which AI models can benefit. However, OCT imaging may not yet have the same level of standardization across different devices and studies, resulting in variability that can challenge model performance [69].

In general, DL models demonstrated less robust performance in predicting glaucoma progression using fundus and OCT compared to their diagnostic capabilities. Predicting disease progression requires large-scale longitudinal data that track patients over time for good accuracy. For the time being, such datasets are still less prevalent, thus limiting the amount of data available for robust model training [70]. Additionally, while longitudinal data are valuable, modeling temporal data often necessitates complex DL architectures, like recurrent neural networks (RNNs) or long short-term memory (LSTM), which could further affect the model’s performance [71]. For instance, Thakur et al. [72] trained a DL algorithm for predicting glaucoma development prior to disease onset using fundus photography from a longitudinal database. However, the longer the time was before disease onset, the less accurate the model was [72].

Additionally, this meta-analysis included studies that primarily trained DL algorithms for progression prediction using fundus and OCT imaging only. Although these modalities are effective in structural assessments and, therefore, glaucoma diagnosis, they may not reflect the functional deterioration associated with glaucoma progression [73]. Notably, additional studies [74,75,76,77] not included in this meta-analysis (due to being excluded for not meeting the outcome-type criteria) involved transforming fundus structural characteristics data into quantitative functional data, such as VF parameters, based on optic disc photographs or OCT images. Other predictors, such as IOP and central corneal thickness (CCT), could potentially enhance the performance and refinement of diagnostic algorithms by providing additional physiological and biomechanical context to the analysis [78,79]. However, these factors were not included as primary components in the training processes of the included studies. Our study results suggest that although fundus photography and OCT imaging alone can yield significant findings, the lack of robustness indicates that integrating functional assessment data, such as VF measurements, may be essential to further enhance DL models for predicting glaucoma progression.

What are the practical steps to enhance DL model performance then? Attention mechanisms can further enhance such frameworks by enabling the model to focus on critical features from each modality [80]. For instance, an attention-based DL model trained on multimodal inputs might prioritize optic nerve head parameters from fundus images and retinal nerve fiber layer thickness from OCT scans while integrating these with VF defect patterns, thus leveraging both structural and functional markers. Additionally, a practical approach involves developing a dual-stream CNN architecture that processes structural imaging data with functional assessments, therefore allowing the model to capture intricate patterns associated with glaucoma more effectively [81].

Based on this meta-analysis, the superior performance of the internal validations compared to the external validations highlights the important aspects of model training and evaluation. With internal validation, certain studies typically involve testing the model on a subset of the same dataset used for training, often split via methods like k-fold cross-validation [21,24,40]. As a result, the data used for the internal testing might share similar characteristics, such as imaging protocols, patient demographics, and disease prevalence, which can lead to inflated performance metrics [82,83].

Certain studies [37,43,45,48,49] in the meta-analysis employed both internal and external testing with different datasets, with generally lower accuracy in external validation. This highlighted that some DL models were prone to overfitting, in which the algorithms performed well on training data because they had learned both signals and noises, but made generalization difficult [84]. However, future studies are likely to include data from multiple sources, imaging devices, and patient populations during their training stage [43]. Factors such as different imaging devices, settings, and patient populations may affect the model’s performance and its ability to generalize during external testing too [85].

It is also important to compare the performances of these DL algorithms with non-AI-based approaches to assess how DL models can complement or enhance conventional diagnostic techniques. Traditional diagnostic approaches, such as visual field testing and optic nerve head evaluation, also show good diagnostic performance but with more variability across studies [86]. These methods typically yield sensitivity values between 0.75 and 0.85, depending on the stage of glaucoma and the quality of the test [87]. They are often time-consuming, require expert knowledge, and are subject to inter-observer variability. In contrast, our study aligns with previous research that has shown how AI-based tools can quickly analyze fundus and OCT images, providing reliable diagnostic results with good sensitivity and specificity and reducing the need for costly and labor-intensive manual analysis [10,16,88].

One of the limitations of our meta-analysis is the heterogeneity among the included and analyzed studies. Variations in DL architectures, imaging protocols, and patient populations from multiple nations across different continents may contribute to variability in DL performance. Nevertheless, the considerable heterogeneity observed was anticipated due to the large number of included studies and the diverse nature of real-world study designs. To account for this variability, a random-effects model was employed in the analysis. While the inclusion of progression prediction studies is a strength of this meta-analysis, the lack of longitudinal datasets and the reliance on imaging alone to capture quantitative characteristics signifying progression may have resulted in less robust findings. To more accurately reflect the state of deep learning applications in glaucoma progression prediction, a greater number of datasets incorporating multimodal data—including visual field assessments—are needed in the literature.

In conclusion, this meta-analysis demonstrates that DL algorithms achieve optimal performance in diagnosing glaucoma and predicting its progression using fundus photography and OCT imaging. However, the results also reflect real-world observations where external testing tends to have lower accuracy than internal testing due to the greater variability in datasets, reference standards, and clinical settings. The challenges for DL algorithms in glaucoma include the need for high-quality annotated datasets for training, the potential for overfitting, and the need for clinical validation in real-world settings. Despite these challenges, this study adds to the growing body of evidence supporting the current integration of AI-based tools to enhance glaucoma diagnosis and progression predictions, though they should not replace clinical judgment.

## Figures and Tables

**Table 1:** Characteristics of selected studies (datasets) used in glaucoma diagnosis.

Study (Year)	Country/ Region	DL Algorithm	Data Type	Testing Data Sets	Total Testing Data Size (Images)	Sensitivity	Specificity	AUC	Internal/ External Validation
Al-Aswad (2019) [19]	United States	ResNet50	Fundus imaging	Singapore Malay Eye Study	110	0.84	0.88	0.93	External
Asaoka (2018) [20]	Japan	Deep Learning (CNN) with Transfer Learning	OCT	Private	196	0.82	0.94	0.94	Internal
Bajwa (2019) [21]	Europe	RCNN	Fundus imaging	ORIGA, HRF, OCT, CFI	551	0.81	0.70	0.87	External
Chakrabarty (2016) [22]	India	Image vector	Fundus imaging	Private	314	0.72	0.72	0.79	External
Chang (2021) [23]	Korea	ResNet50	Fundus imaging	Private	605	0.79	0.90	0.90	Internal
Christopher (2018) [24]	USA	CNN	Fundus imaging	The African Descent and Glaucoma Evaluation Study(ADAGES) and University of California, San Diego (UCSD)	1482	0.84	0.83	0.91	External
Civit-Masot (2020) [25]	Spain	MobileNet V2	Fundus imaging	RIM-ONE	232	0.89	0.82	0.83	Internal
Diaz-Pinto (2019) [26]	Spain	CNN	Fundus imaging	ACRIMA	1707	0.93	0.86	0.96	External
Fu (2020) [27]	China	U-Net network	Fundus imaging	ORIGA, Singapore Chinese Eye Study (SCES), Singapore Indian Eye Study (SINDI)	1676	0.85	0.84	0.84	External
Gomez-Valverd (2019) [28]	Spain	CNN	Fundus imaging	RIM-ONE, ESPERANZA, DRISHTI-G	579	0.87	0.89	0.94	Internal
Hemelings (2023) [29]	Multiple	G-risk regression model	Fundus imaging	BMES, GHS, AIROGS, ORIGA, REFUGE1, ODIR, REFUGE2, GAMMA, RIM-ONEr3, RIM-ONE DL, ACRIMA, PAPILA	3554	0.87	0.95	0.98	External
Kashyap (2022) [30]	Multiple	Dense-Net 201	Fundus imaging	Singapore Malay Eye Study	162	0.95	0.95	0.95	Internal
Kausu (2018) [31]	India	CNN	Fundus imaging	Private	86	0.97	0.98	0.98	Internal
Kim (2020) [32]	Korea	CNN	OCT	Private	425	0.98	0.90	0.98	Internal
Ko (2020) [33]	Taiwan	CNN	Fundus imaging	Private	187	0.96	0.94	0.99	Internal
Lee (2019) [34]	Korea	CNN	OCT	Private	200	0.93	0.84	0.94	Internal
Lee (2020) [35]	Korea	CNN	OCT	Private	85	0.95	1.00	0.99	Internal
Lee’ (2020) [36]	Korea	CNN	OCT	Private	81	0.97	0.93	0.99	Internal
Li (2022) [37]	China	DiagnoseNet	Fundus imaging	Private A	2866	0.89	0.83	0.94	External
				Private B	155	0.92	0.71	0.91	External
Li (2021) [38]	Japan and China	InceptionResNetV2	Fundus imaging	Chinese Medical Alliance for Artificial Intelligence (CMAAI)	783	0.97	0.98	0.99	Internal
Li (2020) [39]	China	AG-CNN	Fundus imaging	LAG database	832	0.95	0.97	0.98	Internal
Li’ (2020) [40]	China	ResNet101	Fundus imaging	Private	3481	0.96	0.93	0.99	Internal
Lin (2022) [41]	USA	CNN	Fundus imaging	OHTS	37339	0.90	0.90	0.90	External
Liu (2018) [42]	Australia	ResNet50	Fundus imaging	RIM-ONE and Sydney-based images	800	0.89	0.97	0.97	Internal
				High Resolution Fundus (HRF) database	30	0.87	0.87	0.89	External
Liu (2019) [43]	China	GD-CNN	Fundus imaging	CSGA					Internal
				Bejing Tongren Hospital	20,466	0.96	0.97	0.99	External
				Peking Third Hospital	12,718	0.96	0.96	0.99	External
				Harbin Medical University	9305	0.94	0.96	0.99	External
				Handan Eye Study	29,676	0.91	0.93	0.96	External
				Hamilton Glaucoma Center	7877	0.87	0.81	0.92	External
				Websites	884	0.82	0.71	0.82	External
Liu’ (2019) [44]	China	Semi-supervised Conditional GANs	Fundus imaging	ORIGA, REFUGE, RIM-ONE, DRISHTI-GS	100	0.73	0.80	0.86	External
Maccormick (2019) [45]	Singapore	Spatial probalistic model	Fundus imaging	ORIGA	325	1.00	0.98	0.99	Internal
				RIM-ONE	159	0.90	0.79	0.91	External
Martins (2020) [46]	Portugal	CNN	Fundus imaging	ORIGA, Drishti-GS, RIM-ONE, iChallenge, RIGA	2482	0.85	0.88	0.93	Internal
Meideros (2021) [47]	USA	CNN	OCT	Duke Glaucoma Repository	6292	0.76	0.95	0.94	Internal
Noury (2022) [48]	Multiple	3D Deep Learning Algorithm	OCT	Stanford	694	0.86	0.78	0.91	Internal
				Hong Kong	1625	0.73	0.73	0.80	External
				India	672	0.93	0.71	0.94	External
				Nepal	380	0.79	0.79	0.90	External
Phene (2019) [49]	Multiple	CNN	Fundus imaging	EyePACS, UK Biobank, AREDS etc.	1205	0.80	0.90	0.95	Internal
				VA Eye Clinics	9642	0.80	0.85	0.86	External
				India	346	0.85	0.80	0.92	External
Raghavendra (2019) [50]	India	Two Layer Sparse Autoencoder	Fundus imaging	Manipal College	428	0.95	0.95	0.95	Internal
Ran (2019) [51]	Hong Kong	CNN	Fundus imaging	Hong Kong Eye Hospital	975	0.78	0.86	0.91	External
Rogers (2019) [52]	UK, Europe	CNN	Fundus imaging	European Optic Disc Assessment Study (EODAT)	94	0.81	0.86	0.87	External
Shibata (2018) [53]	Japan	CNN	Fundus imaging	Matsue Red Cross Hospital	110	0.97	0.95	0.97	Internal
Singh (2021) [54]	India	SVM	OCT	Mendeley and private dataset	140	1.00	0.85	0.97	Internal
Soorya (2021) [55]	India	Cloud-based system	Fundus imaging	Private	364	0.86	0.96	0.91	Internal
Thompson (2020) [56]	USA	CNN	OCT	Duke Glaucoma Repository	6340	0.81	0.95	0.96	Internal
Wu (2022) [57]	Taiwan	SVM	OCT	KaoHsiung Medical University Hospital and Fu-Jen Catholic Hospital	752	0.85	0.70	0.82	Internal
Xu (2021) [58]	China	Hierarchical Deep Learning System (HDLS)	Fundus imaging	Bejing Tongren Hospital	400	0.96	0.94	0.95	Internal
Yang (2020) [59]	Korea	CNN	Fundus imaging	Seoul National University Hospital	2675	0.86	0.99	0.95	Internal
Zheng (2020) [60]	China	CNN	OCT	Shantou and Hong Kong	102	0.98	0.90	0.94	Internal

Fundus imaging indicates retinal fundus image. OCT: optical coherence tomography. CNN: convolutional neural network. Private: authors used their data or not publicly accessed data to train and evaluate the model. Total testing data size: total number of images used to evaluate, test, or validate the model. Internal validation: Validating/testing and training data came from the same database(s). External validation: Validating/testing and training data came from different databases.

**Table 2:** Characteristics of selected articles (datasets) used in glaucoma progression prediction.

Study (Year)	Country/ Region	DL Algorithm	Data Type	Testing Data Sets	Total Testing Data Size (Eyes)	Sensitivity	Specificity	AUC	Internal/ External Validation
Brown (2023) [61]	United States	Object detection model	Fundus	New York Eye and Ear Infirmary of Mount Sinai (NYEE- MSSM), Ocular Hypertension Treatment Study (OHTS), Thessaloniki Eye Study (TES), Massachusetts Eye and Ear (MEE), Glaucomatous Optic Neuropathy Evaluation (GONE)	165	0.97	0.73	0.94	Internal
Ha (2024) [62]	Korea	XGBoost, Random Forest, Gradient Boosting	Fundus	Private	210	0.98	0.98	0.99	Internal
Hussain (2023) [63]	Multiple	Multimodal deep learning with GAN	OCT	Vilnius University Hospital Santaros Klinikos	21	0.73	0.73	0.68	Internal
Li (2022) [37]	China	DiagnoseNet	Fundus	Private A	337	0.82	0.59	0.87	External
				Private B	513	0.81	0.74	0.88	External
Mandal (2024) [64]	United States	Noise-Positive Unlabeled	OCT	Bascom Palmer Eye Institute and Duke	446	0.50	0.95	0.50	Internal
Mariotonni (2023) [65]	United States	CNN	OCT	Duke Glaucoma Registry	924	0.87	0.86	0.94	Internal
Meideros (2021) [66]	United States	ResNet50	OCT	Duke Glaucoma Registry	1147	0.85	0.90	0.96	Internal

Fundus imaging indicates retinal fundus image. OCT: optical coherence tomography. CNN: convolutional neural network. Private: authors used their data or not publicly accessed data to train and evaluate the model. Total testing data size: total number of images used to evaluate, test, or validate the model. Internal validation: validating/testing and training data came from the same database(s). External validation: validating/testing and training data came from different databases.

**Table 3:** Summary estimates for overall AI performance in glaucoma detection and subgroup analyses.

Variables	No. of Tested Images	Sensitivity (95% CI)	Specificity (95% CI)	LR+ (95% CI)	LR− (95% CI)	AUROC (95% CI)
Fundus pictures						
All data	194,259	0.92 (0.89–0.94)	0.93 (0.90–0.95)	12.99 (9.23–18.30)	0.09 (0.07– 0.12)	0.90 (0.88–0.92)
Internal	124,552	0.93 (0.91–0.95)	0.95 (0.93–0.97)	18.77 (13.09–26.91)	0.07 (0.05–0.10)	0.91 (0.90–0.93)
External	69,707	0.86 (0.82–0.93)	0.88 (0.80–0.93)	7.259 (4.14–12.73)	0.13 (0.08–0.22)	0.88 (0.86–0.91)
OCT						
All data	17,882	0.90 (0.84–0.94)	0.87 (0.81–0.91)	6.87 (4.57–10.33)	0.11 (0.07–0.19)	0.86 (0.83–0.90)
Internal	15,009	0.93 (0.85–0.96)	0.89 (0.83–0.93)	8.46 (5.23–13.68)	0.08 (0.04–0.17)	0.89 (0.85–0.93)
External	2873	0.83 (0.73–0.90)	0.80 (0.68–0.88)	4.20 (2.56–6.89)	0.21 (0.13–0.34)	0.80 (0.75–0.86)

Abbreviations: AUROC = area under receiver operator characteristic; OCT = optical coherence tomography; LR+ = positive likelihood ratio; LR− = negative likelihood ratio.

**Table 4:** Summary estimates for overall AI performance in glaucoma progression prediction and subgroup analyses.

Variables	No. of Tested Eyes	Sensitivity (95% CI)	Specificity (95% CI)	LR+ (95% CI)	LR− (95% CI)	AUROC (95% CI)
Fundus pictures						
All data	841	0.89 (0.78–0.95)	0.77 (0.64–0.87)	3.88 (2.31–6.51)	0.14 (0.07–0.30)	0.91 (0.85–0.97)
Internal	375	0.95 (0.82–0.99)	0.88 (0.60–0.97)	7.87 (1.97–31.48)	0.06 (0.02–0.23)	0.94 (0.84–1.00)
External	466	0.81 (0.77–0.85)	0.67 (0.51–0.79)	2.44 (2.18–2.73)	0.28 (0.23–0.34)	0.88 (0.84–0.92)
OCT						
Internal	2538	0.74 (0.51–0.89)	0.93 (0.88–0.96)	10.09 (7.60–13.39)	0.28 (0.14–0.57)	0.90 (0.84–0.95)

Abbreviations: AUROC = area under receiver operator characteristic; OCT = optical coherence tomography; LR+ = positive likelihood ratio; LR− = negative likelihood ratio.

## Data Availability

Data sharing is not applicable to this article, as no new data were created in this study. All data supporting the findings are available from the original publications cited in the reference list.

## Supplementary Materials

The following supporting information can be downloaded at https://www.mdpi.com/article/10.3390/biomedicines13020420/s1, Supplementary Figure S1: Quality assessment of included studies using the QUADAS-2 tool; Supplementary Figure S2: Graphical presentation of quality assessment using the QUADAS-2 tool; Supplementary Figure S3: Deeks’ funnel plot asymmetry test; Supplementary Table S1: Statistical analyses with DeLong’s test to compare all sensitivities, specificities, likelihood ratios, and AUROCs.
