# Supplementary material for: Deep Learning in Glaucoma Detection and Progression Prediction: A Systematic Review and Meta-Analysis
Source: Biomedicines. 2025 Feb 10;13(2):420. doi: 10.3390/biomedicines13020420 (PMC11852503; doi:10.3390/biomedicines13020420)
Supplement: Supplementary file 1 [file biomedicines-13-00420-s001.zip › Supplementary Materials.pdf]

DETAILED SEARCH STRATEGY FOR STUDIES ENTRY

PUBMED:

((((((((((glaucoma) OR (open angle glaucoma)) OR (primary open angle glaucoma)) OR (ocular hypertension)) OR (normal tension glaucoma)) OR (glaucoma suspect)) OR (angle closure glaucoma)) OR (glaucomatous optic neuropathy)) AND ((journalarticle[Filter]) AND (fft[Filter]))) AND ((((((((artificial intelligence) OR (machine learning)) OR (deep learning)) OR (transfer learning)) OR (neural network)) OR (algorithms)) OR (Supervised Machine Learning)) OR (Unsupervised Machine Learning))) AND (((glaucoma progression) OR (glaucoma diagnosis))) Filters: Full text, Journal Article

EMBASE:

#3: #1 AND #2 AND ('article'/it OR 'article in press'/it)

#2: 'artificial intelligence'/exp OR 'artificial intelligence' OR (artificial AND ('intelligence'/exp OR intelligence)) OR 'machine learning'/exp OR 'machine learning' OR (('machine'/exp OR machine) AND ('learning'/exp OR learning)) OR 'deep learning'/exp OR 'deep learning' OR (deep AND ('learning'/exp OR learning)) OR 'transfer learning'/exp OR 'transfer learning' OR (('transfer'/exp OR transfer) AND ('learning'/exp OR learning)) OR 'neural network'/exp OR 'neural network' OR (neural AND ('network'/exp OR network)) OR 'algorithms'/exp OR algorithms OR 'supervised machine learning'/exp OR 'supervised machine learning' OR (supervised AND ('machine'/exp OR machine) AND ('learning'/exp OR learning)) OR 'unsupervised machine learning'/exp OR 'unsupervised machine learning' OR (unsupervised AND ('machine'/exp OR machine) AND ('learning'/exp OR learning))

#1: 'glaucoma'/exp OR glaucoma OR 'open angle glaucoma'/exp OR 'open angle glaucoma' OR (open AND angle AND ('glaucoma'/exp OR glaucoma)) OR 'primary open angle glaucoma'/exp OR 'primary open angle glaucoma' OR (primary AND open AND angle AND ('glaucoma'/exp OR glaucoma)) OR 'ocular hypertension'/exp OR 'ocular hypertension' OR (ocular AND ('hypertension'/exp OR hypertension)) OR 'normal tension glaucoma'/exp OR 'normal tension glaucoma' OR (normal AND ('tension'/exp OR tension) AND ('glaucoma'/exp OR glaucoma)) OR 'glaucoma suspect' OR (('glaucoma'/exp OR glaucoma) AND suspect) OR 'angle closure glaucoma'/exp OR 'angle closure glaucoma' OR (angle AND closure AND ('glaucoma'/exp OR glaucoma)) OR 'glaucomatous optic neuropathy'/exp OR 'glaucomatous optic neuropathy' OR (glaucomatous AND optic AND ('neuropathy'/exp OR neuropathy)) OR 'glaucoma diagnosis'/exp OR 'glaucoma diagnosis' OR 'glaucoma progression'/exp OR 'glaucoma progression' OR (glaucoma AND diagnosis) OR (glaucoma AND progression) OR (glaucoma AND screening)

WEB OF SCIENCE:

TS=("glaucoma" OR "open angle glaucoma" OR "primary open angle glaucoma" OR "ocular hypertension" OR "normal tension glaucoma" OR "glaucoma suspect" OR "angle closure glaucoma" OR "glaucomatous optic neuropathy" OR "glaucoma diagnosis" OR "glaucoma progression" OR ("glaucoma" AND "diagnosis") OR ("glaucoma" AND "progression")) AND TS=("artificial intelligence" OR "machine learning" OR "deep learning" OR "transfer learning" OR "neural network" OR "algorithms" OR "supervised machine learning" OR "unsupervised machine learning")
