# Supplementary figures and images for: Deep Learning in Glaucoma Detection and Progression Prediction: A Systematic Review and Meta-Analysis
Source: Biomedicines. 2025 Feb 10;13(2):420. doi: 10.3390/biomedicines13020420 (PMC11852503; doi:10.3390/biomedicines13020420)

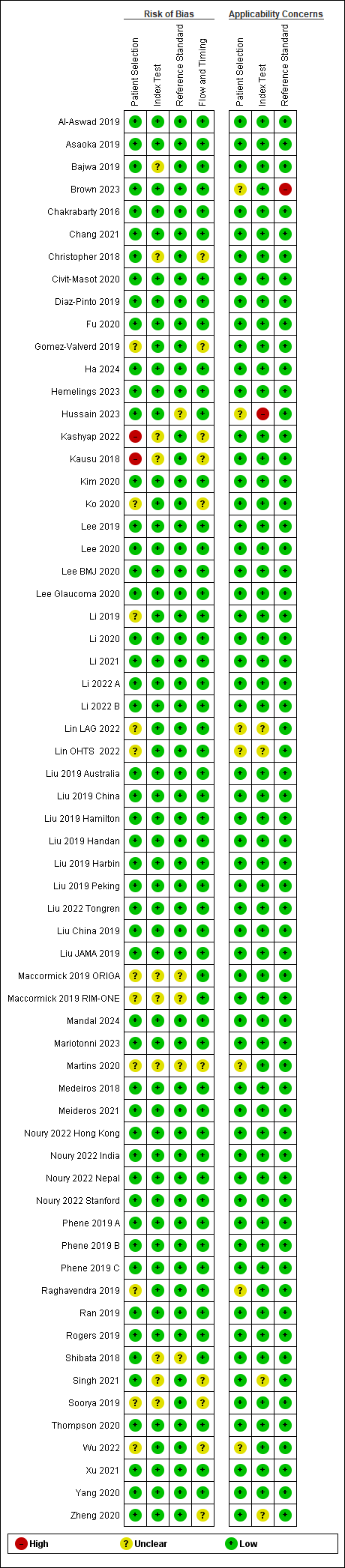

Supplement: Supplementary file 1 [file biomedicines-13-00420-s001.zip › Supplementary Figure S1.png.png]

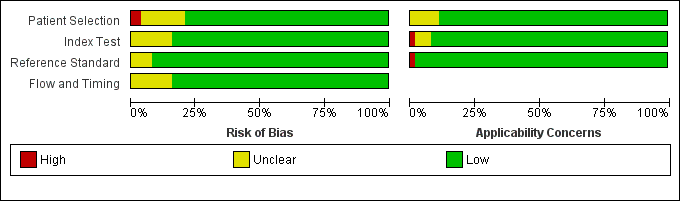

Supplement: Supplementary file 1 [file biomedicines-13-00420-s001.zip › Supplementary Figure S2.png.png]

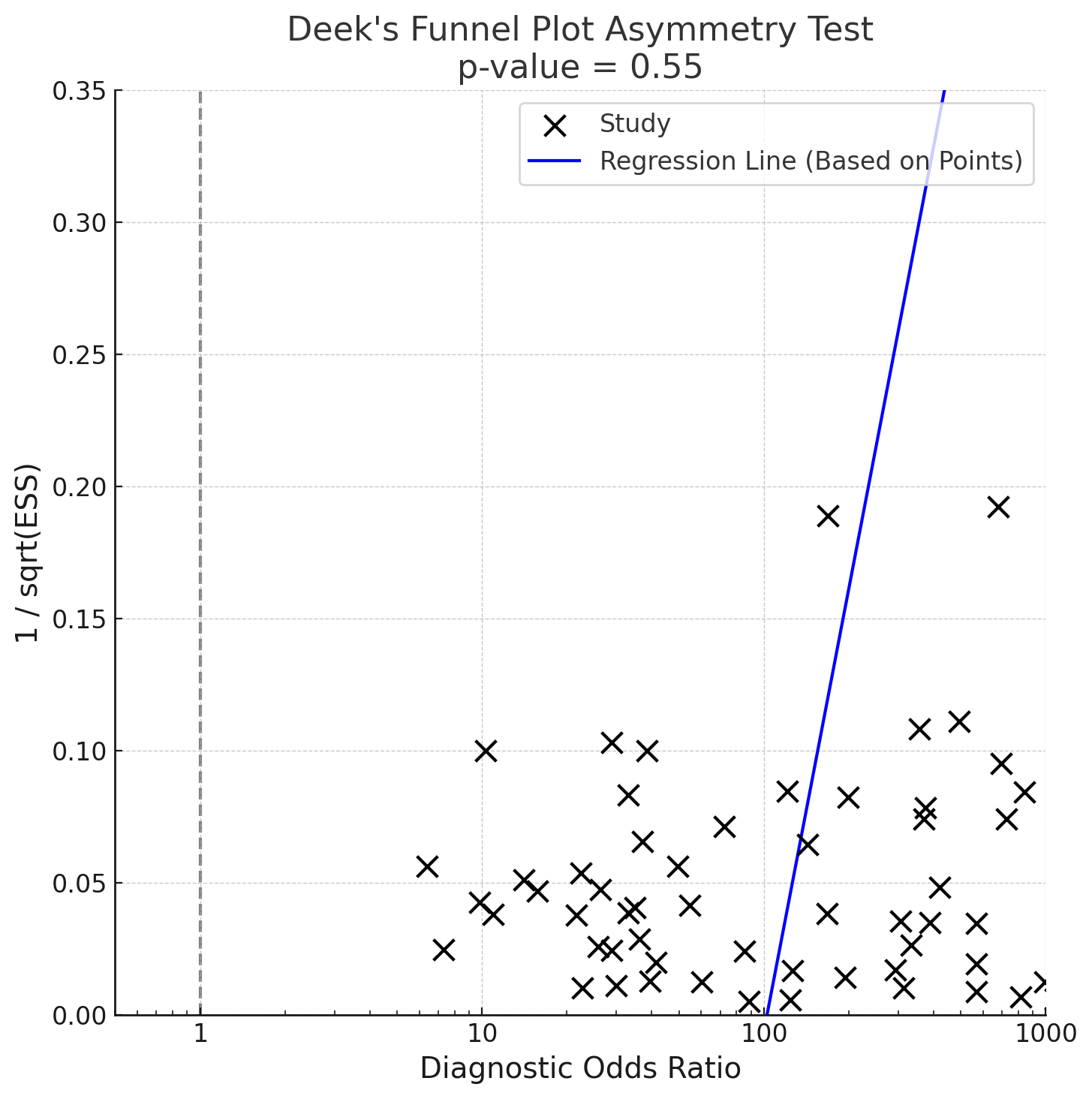

Supplement: Supplementary file 1 [file biomedicines-13-00420-s001.zip › Supplementary Figure S3.png.png]
